# Echoes of the Past: Hereditarianism and *A Troublesome Inheritance*

**DOI:** 10.1371/journal.pgen.1004817

**Published:** 2014-12-11

**Authors:** Marcus Feldman

**Affiliations:** Stanford University, Stanford, California, United States of America; The University of North Carolina at Chapel Hill, United States of America

There has been considerable debate about the representation of human population genetics in a recent book by journalist Nicholas Wade: *A Troublesome Inheritance*. A letter http://cehg.stanford.edu/letter-from-population-geneticists/signed by 143 scientists, including 16 members of the *PLOS Genetics* Editorial Board, and PLOS founder Mike Eisen, criticized the book in the *New York Times Book Review* on August 8, 2014. As there is widespread interest in this issue from our Editorial Board, we have invited Marcus Feldman to publish his book review in *PLOS Genetics*.

In 1969, Arthur Jensen ignited a decades-long debate when he wrote that it is a “not unreasonable hypothesis that genetic factors are strongly implicated in the average Negro–white intelligence difference” [1]. From this he inferred that educational interventions in communities whose members have lower measured intelligence quotient (IQ) could not succeed.

The errors in Jensen's choice of data [2] and statistical methods used to compute a heritability of about 80% for measured IQ were pointed out by numerous geneticists and statisticians. 25 years after Jensen's incendiary paper, Herrnstein and Murray's book, *The Bell Curve* [3], drew inferences similar to Jensen's that differences among races and social classes in IQ were genetically based. *The Bell Curve* elicited a flood of strong criticisms of the data used, the statistical analyses, and the policy inferences [4]. Much of the criticism of Jensen and Herrnstein and Murray centers on their interpretation of heritability of IQ. In 1975, Richard Lewontin and I [5] stressed the failure of the heritability statistic to do what these authors claim; namely, to show that IQ is largely genetically determined and hence that traits related to IQ, such as educational or economic success, would be impervious to environmental intervention.

As pointed out by Nicholas Wade in the first half of *A Troublesome Inheritance*, we are now in a genomic age, where individual differences at the level of DNA can be detected. The early chapters present a hodgepodge of historical ideas about race, aggression, and genetics. On page 57, Wade gives an inkling of what will come in the last half of the book: “important aspects of human social behavior are shaped by the genes” and “these behavior traits are likely to vary from one race to another, sometimes significantly so” [6].

Whereas inferences on the causes of human behavioral variation referred to above were based on correlations between relatives, on pages 97–99 Wade develops his arguments for the genetic basis of social behaviors in the second half of *A Troubled Inheritance* from results on worldwide variation in DNA polymorphisms, namely microsatellite polymorphisms (The Rosenberg-Feldman studies) [7], [8] and single nucleotide polymorphisms (another Stanford study) [9], from the Human Genome Diversity Panel [10]. Here, as in his previous journalism about these studies, Wade exhibits a complete lack of understanding of their implications. For example, he does not mention the finding, stressed in both studies, that only 5%–10% of the worldwide genomic variation is between continental groups, while the vast majority is between individuals within populations.

Using data from 15 protein genes, R. C. Lewontin in 1972 [11] was the first to point out that the overwhelming majority of human genotypic variation is within populations, and that continental “races” differed little genetically. 25 years later, Barbujani et al. [12] came to the same conclusion from their study of 109 DNA markers. Wade (page 120) criticizes Lewontin's conclusion that “racial classification is now seen to be of virtually no genetic or taxonomic significance” as representing Lewontin's “political stake in the issue.”

From the data and analyses of worldwide molecular genomic variation, Richard Lewontin and I amplified the conclusions of Lewontin and Barbujani et al. as follows: “The repeated and consistent results on the apportionment of genetic diversity…show that the genes underlying the phenotypic differences used to assign race categories are atypical of the genome in general and are not a reliable index to the amount of genetic differentiation between groups. Thus, racial assignment loses any general biological interest. For the human species, race assignment of individuals does not carry with it any general implication about genetic differentiation” [13]. The increased resolution on patterns of human variation that we now have has enabled us to understand a great deal about human migration, admixture, population size, and natural selection. However, it has not told us that the earlier studies underestimated the biological reality of race.

Even though the between-continent fraction of genetic variation is small, as the reader discovers on leaving the first half of *A Troublesome Inheritance*, Wade's erroneous interpretation of its significance for racial differences becomes the basis for his entry into the “speculative arena at the interface of history, economics, and human evolution” (page 15). In the second half of the book, he claims that differences among continents in economic development, social institutions, and social behaviors are based in genetics. This classic correlation–causation error cannot be excused on the grounds that Wade is just speculating; continents can be distinguished genetically; they also have different economic and social histories. One cannot conclude, as Wade does, that the former causes the latter.

The first paragraph of chapter seven summarizes Wade's process of inference: “Each of the major civilizations has developed institutions appropriate for its circumstances and survival. But these institutions, though heavily imbued with cultural traditions, rest on a bedrock of genetically shaped human behavior; and when a civilization produces a distinctive set of institutions that endures for many generations, that is the sign of a supporting suite of variations in the genes that influence human social behavior.” I will focus on two of the studies invoked by Wade to justify his totally unfounded claims that differences in the societies of different continents (which he terms “races” even though in a biological sense they are not understood as such) are due to their genetic differences.

The first is by Gregory Clark, an economic historian who studies changes in interpersonal violence, literacy, the propensity to save, and the propensity to work in the English population from 1200 CE to 1800 CE [14]. As Wade puts it on page 154, during this period “the nature of the people had changed.” Between 1200 and 1800, “these behavioral changes in the English population…gradually transformed a violent and undisciplined peasant population into an efficient and productive workforce.” On pages 159–161, Wade explains how this happened. “Clark has uncovered the simple genetic mechanism…the rich had more surviving children than the poor.” And further, “Most children of the rich had to sink in the social scale,” and as a result, “their social descent had the far reaching genetic consequence that they carried with them the same behaviors that had made their parents rich.”

Against the argument that changing culture may have been involved in the 600-year process, Wade states (page 160) that these “behaviors emerged gradually over several centuries, a time course more typical of an evolutionary change than a cultural change.” To justify his claim that 600 years is enough time to have produced “significant changes in social behavior” (page 161) of the English, Wade leans on experiments by Belyaev, who artificially selected silver foxes for tameness. The strength of this selection was extreme: “typically not more than 4 or 5 percent of male offspring and about 20 percent of female offspring have been allowed to breed” [15]. The strongest natural selection on humans is orders of magnitude weaker than this “sufficiently intense” artificial selection imposed on the foxes (page 161). Few evolutionists would agree that 600 years, that is, about 25 generations, is long enough for such significant behavioral changes to be due to human genetic evolution; here and elsewhere in the book, Wade uses “evolutionary” where it is obvious that he means “genetic.” “Ingrained” is another euphemism he occasionally uses. For example, on page 177, “Tribal behavior is more deeply ingrained than are mere cultural prescriptions. Its longevity and stability point strongly to a genetic basis.” Galton and Pearson would have approved of Wade's espousal of a genetic basis for class differences; there is more than a whiff of eugenics here.

Wade devotes almost four pages of chapter seven, the longest chapter in the book, to IQ. After claiming (page 190) that “intelligence is almost certainly under genetic influence,” he goes on to discuss the relationship between wealth and IQ and invokes the work of Richard Lynn and Tatu Vanhanen, in particular their book *IQ and Global Inequality* [16].

Lynn is known for his work as an associate editor of *The Mankind Quarterly*, described by the famous psychologist Leon Kamin as a “vulgarly racist” [17] journal. Lynn's 1991 paper on IQ of Africans is described by Kamin as “truly venomous racism, combined with scandalous disregard for scientific objectivity” [17]. In 2002, Lynn wrote the nonsensical statement: “The conclusion that there is a true association between skin color and IQ is consistent with the hypothesis that genetic factors are partly responsible for the black–white difference in intelligence…the evidence that a statistically significant correlation is present confirms the genetic hypothesis” [18]. In placing so much emphasis on Lynn's work (which was strongly supported by the notorious Pioneer Fund, which also supported William Shockley and was chaired by an even more notorious scholar, J. Philippe Rushton), Wade has apparently chosen to ignore important studies on IQ and environment such as those by Brooks–Gunn et al. [19] and Turkheimer et al. [20]. Brooks–Gunn found that “adjustments for economic and social differences in the lives of black and white children all but eliminate differences in the IQ scores between the two groups,” suggesting that socioeconomic status (SES) might be an important contributor to high heritability estimates. In the same vein, Turkheimer et al. found that heritability of IQ depended strongly on SES: there was a high heritability in higher SES environments, but not in low SES environments. By omitting reference to such studies that find very strong environmental contributions to IQ, while focusing on Richard Lynn, the book takes a very hereditarian stance.

Wade gives the appearance (page 192) of care in interpreting Lynn and Vanhanen: “It is hard to know which way the arrow of causality may be pointing, whether higher IQ makes a nation wealthier or whether a wealthy nation enables its citizens to do better on IQ tests.” However, from his statement (page 203) about “the strong heritability of intelligence” and his belief (page 160, referring back to Clark) that in England “the children of the rich carried with them inheritance for the same behaviors that made their parents rich,” we can only assume that Wade believes there is a genetic basis for both IQ and wealth. His “arrow of causality” has two points, with genetics responsible for both IQ and wealth.

This section of the book is redolent of the claims of Jensen, as well as Herrnstein and Murray, mentioned at the beginning of this review. It also harks back to claims by Taubman [21] in the 1970s, based on correlations between relatives, that variation between individuals in wealth has a strong genetic basis. It is most informative to compare Goldberger's 1977 [22] criticism of Taubman's analysis with related negative evaluations of studies on heritability of IQ. By invoking Richard Lynn on racial variation in IQ and wealth, Wade departs from his “speculative arena,” leaving us to infer that he is a devout hereditarian.

Wade goes even further than proposing a genetic basis for continental variation in wealth; he would have us believe that differences in economic and political institutions among populations have a genetic basis. On page 196, he criticizes the book *Why Nations Fail* by Acemoglu and Robinson [23] because “they have ruled out the obvious possibility that variations in human behavior are the cause of good or bad institutions.” Variation in institutions is why “a part of the world has grown steadily and vastly richer over the past 300 years.” He concludes that a reasonable explanation for this variation “is available in terms of human evolution.”

Wade is using “evolution” here to mean the production and maintenance of genetic differences, and “variations in human behavior” is his euphemism for racial, and hence (in his understanding) genetic differences. He appears to backtrack slightly in the final chapter (pages 240–241), where he poses the paradox “that people as individuals are so similar yet human societies differ so copiously.” His resolution of the paradox is that these societal differences “stem from the quite minor variations in human social behavior…that have evolved within each race during its geographical and historical experience.” Again, “evolved” must be understood in genetic terms: it is “because of their institutions—which are largely cultural edifices resting on a base of genetically shaped social behaviors—that the societies of the West and East Asia are so different.”

We can juxtapose Wade's conclusions on the genetic basis of racial differences in wealth, economies, and institutions with those of Ashraf and Galor on a similar topic. Their claim was that the high and low molecular genetic diversity characteristic of African and Native American populations, respectively, “have been detrimental for the development of these regions,” while “the intermediate levels of diversity associated with European and Asian populations have been conducive for development” [24]. Wade's use of worldwide patterns of human molecular genetic variation to define races and his inference that genetic variation between races explains their economic differences are qualitatively similar to Ashraf and Galor's thesis. Speculation aside, readers of *A Troublesome Inheritance* are advised to heed the admonition by Guedes et al. concerning Ashraf and Galor: “bold claims on the basis of weak data and methods can have profoundly detrimental social and political effects” [25].

Wade's premise is that molecular population genetics has shown sufficient variation between continents to define races. He argues (page 126) that these genetic differences are responsible for differences in individual social behaviors that “undergird” societal institutions, which themselves differ among races. Ironically, the molecular genomic data that have become available in the last fifteen years—the very data that Wade argues we need to bravely acknowledge—explain in terms of admixture, gene flow, demographic change, etc., why human genetic variation is arrayed the way it is. Echoes of the hereditarian arguments about racial difference in IQ and the reductionist arguments of sociobiology and evolutionary psychology resound in *A Troublesome Inheritance*. I have no trouble with the existence of human genetic variation. It is Wade's dangerous interpretation, however speculative, of the meaning of human genetic variation that is indeed troublesome.
